# Safety of Ticagrelor Compared to Clopidogrel in the Contemporary Management Through Invasive or Non-Invasive Strategies of Elderly Patients Presenting with Acute Coronary Syndromes

**DOI:** 10.3390/jcm14165629

**Published:** 2025-08-08

**Authors:** Anum Nazir, Smrthi Shetty Ujjar, Seemi Saba, Neil Ruparelia, Nicos Spyrou, Lampson Fan

**Affiliations:** 1 The Royal Wolverhampton NHS Trust, New Cross Hospital, Wolverhampton WV10 0QP, UKsmrthi.shetty@nhs.net (S.S.U.); seemi.saba@nhs.net (S.S.); 2 Royal Berkshire Hospital, Reading RG1 5AN, UK; neil.ruparelia@nhs.net (N.R.); nicos.spyrou@royalberkshire.nhs.uk (N.S.); 3 University of Birmingham, Birmingham B15 2T, UK

**Keywords:** ACS, ticagrelor, bleeding, elderly patients

## Abstract

**Background:** ESC recommends ticagrelor over clopidogrel for the treatment of acute coronary syndrome (ACS) but the lack of evidence for elderly patients (≥75) and concerns over bleeding has led to significant variability in its use within the UK. Our aim is, therefore, to compare the safety of ticagrelor compared to clopidogrel in real-world elderly patients admitted with ACS and managed either medically or through percutaneous intervention. **Methods:** Unselected elderly patients (≥75) admitted to Royal Berkshire Hospital with ACS (2013–2015) were identified and followed for 1 year. The primary outcomes were bleeding events (TIMI criteria), all-cause mortality, cardiovascular mortality, ischemic stroke, angina, NSTEMI and STEMI. **Results:** A total of 288 patients with ACS were discharged with aspirin and either clopidogrel (137) or ticagrelor (151). In total, 152 of these patients underwent invasive angiography and revascularization. The baseline clinical characteristics and crusade bleeding score were similar between the groups receiving ticagrelor or clopidogrel. There were no significant differences in all-cause mortality (8.8% vs. 10.6%), cardiovascular mortality (2.9% vs. 2.0%), ischemic stroke (0.7% vs. 2.0%), angina (6.6% vs. 5.3%) or STEMI (2.2% vs. 1.3%). Patients on clopidogrel, however, had increased events of NSTEMI compared to ticagrelor (8.0% vs. 2.0%, OR 4.481, 95% CI 1.223–16.42) and overall MI (10.2% vs. 3.3%, *p* = 0.030). No difference was observed in either major (8.8 vs. 8.6%) or minor TIMI bleeding (18.2% vs. 20.5%) and after propensity score matching (minor bleeding *p* = 0.39, major bleeding *p* = 0.76). **Conclusions:** In this real-world analysis, ticagrelor did not increase major or fatal bleeding compared to clopidogrel in elderly patients. In view of the mortality benefit in the large trials, additional cardiovascular benefit of ticagrelor should not be withheld on the basis of age as a perceived risk factor for bleeding in ACS.

## 1. Introduction

Acute coronary syndrome (ACS) remains one of the leading causes of death in the developed world [1]. The elderly (aged ≥ 75) represent a significant proportion of patients presenting with ACS with data from large registries reporting 32% to 43% of non-ST elevation myocardial infarction (NSTEMI) [2] and 24–28% of ST-elevation myocardial infarction (STEMI) [3] admissions. Despite this, the elderly patients are grossly under-represented in clinical trials with over 50% of clinical trials to date having excluded patients older than 75 and only 9% of the total number of patients recruited to studies being ≥75 [2]. Furthermore, there is increasing evidence that an invasive strategy in addition to medical therapy is superior to medical therapy alone in the elderly patients [4], which makes anti-platelet safety even more important with the relative contraindication of premature cessation and risk of stent thrombosis. The recent SENIOR-RITA trial indicated that invasive strategy in patients with NSTEMI significantly reduced the risk of re-infarction and need for subsequent revascularization without any effect on mortality [5].

Based on observational data, age is a major risk factor for the occurrence of major or life-threatening bleeding [6,7]. In addition, elderly patients often have a number of co-morbidities including renal impairment that contribute to an increased risk of bleeding [2,3]. In one cohort study, patients >75 years of age had almost a 3-fold increase in major, fatal or disabling bleed as defined by the CURE criteria [8], over 10 years on single anti-platelet agent compared to patients under the age of 75 [9]. This observation was further supported by data from the TIMI-TRITON 38 trial, where patients aged > 75 treated with prasugrel (as opposed to clopidogrel) did not derive the same benefit as younger patients with regards to Major Adverse Cardiac and Cerebrovascular Events (MACCE) during the follow-up period and suffered more bleeding. As a consequence, its use is not recommended in this patient group, although this has changed in the recent ESC NSTEMI guideline for patients proceeding to PCI [10,11].

Ticagrelor, a potent reversible P2Y12 inhibitor, has been recommended in the latest European Society of Cardiology (ESC) guidelines due to its small but significant mortality benefit when compared to clopidogrel [11,12]. Moreover, a recent propensity-matched large registry from Germany demonstrated a significant reduction in MACCE with ticagrelor when compared to clopidogrel in elderly patients presenting with STEMI [13]. The increased anti-platelet inhibition, however, also results in significantly increased non-CABG-related bleeding. Appropriate concern with regards to bleeding associated with the more potent ticagrelor and paucity of evidence supporting benefit of this intervention in the elderly population results in wide variability in its use and may deprive them of potential significant benefits [14]. The aim of this study was therefore to evaluate 12-month clinical outcomes in an unselected contemporary elderly patient group who were treated with revascularization (where appropriate), secondary preventative measures and discharged on either clopidogrel or ticagrelor in addition to aspirin following emergency presentation with ACS.

## 2. Materials and Methods

### 2.1. Study Population

All patients ≥ 75 presenting to Royal Berkshire Hospital between 2013 and 2015 with ACS who had an indication for dual anti-platelet therapy were retrospectively identified and included by reviewing the Myocardial Ischemia National Audit Project (MINAP) registry. MINAP is a mandatory national registry established in 1999 to examine the quality of management of heart attacks within the UK. Patients that additionally required treatment with oral anti-coagulation (vitamin K antagonist or direct oral anti-coagulant) or those that underwent emergent CABG were excluded from the study. ACS was defined by the ESC guideline [15].

### 2.2. Clinical Management

All patients were treated in accordance with guidelines [15]. Specifically, they were treated with aspirin and an additional anti-platelet in addition to secondary preventative measures. When thought to be clinically indicated, patients proceeded to invasive angiography and revascularization within 48 h of emergency admission.

Ticagrelor was introduced to the Trust in 2014 so patients with ACS all received aspirin in addition to clopidogrel (300 mg loading followed by 75 mg daily) until 2014 and aspirin in addition to ticagrelor (180 mg loading followed by 90 mg twice daily) thereafter, unless the clinician’s preference led to the use of clopidogrel.

### 2.3. Clinical Follow-Up

Follow up data was obtained via clinical visits, diagnosis codes and clinical summary. Mortality data was obtained from the death certificate. Study endpoints were emergency admission with angina, NSTEMI, STEMI, ischemic stroke, major and minor bleeding as defined by the TIMI criteria [16], all-cause mortality and cardiovascular mortality. Angina, NSTEMI and STEMI were defined according to the ESC guideline [15]. Ischemic stroke was defined as focal loss of neurologic function with symptoms lasting at least 24 h or leading to death [12]. Bleeding risk was assessed using the CRUSADE score [7,15].

### 2.4. Statistics

Baseline characteristics were presented as either mean (±standard deviation) or percentage of the total. Continuous variables were tested for normality and data with skewness between −0.5 and +0.5 was considered normal. Normal data was compared using Student’s *t*-test. Non-normal data was compared using the Mann–Whitney test. Categorical variables were compared using Fisher’s exact test. Nominal 12-month outcomes were expressed as odd’s ratio with 95% relative confidence interval. A propensity score for the probability of assignment to ticagrelor vs. clopidogrel was derived using a multivariate regression model in SPSS v23. Variables included were age, sex, clinical presentation and Crusade score. One-to-one propensity matching without replacement and nearest neighbor matching was performed. All tests were 2-sided and *p* < 0.05 was considered to be statistically significant.

## 3. Results

A total of 288 eligible patients were included in the study (Supplementary Figure S1) and their baseline characteristics are summarized in Table 1 and in Supplementary Tables S1 and S2) following PSM. The mean age was 80 ± 5 and as expected in this demographic group, there was a high prevalence of co-morbidities such as hypertension, diabetes, hypercholesterolemia and cerebral vascular disease.

### 3.1. Presentation and Clinical Management

The ticagrelor group had a higher proportion of STEMI compared to the clopidogrel group on presentation (43.0% vs. 31.4% *p* = 0.051) and more anterior territory infarcts (33.8% vs. 25.5%, *p* = 0.157).

A total of 152 patients underwent invasive angiography and revascularization. All patients were discharged with DAPT regardless of revascularization strategy, with 137 patients discharged on clopidogrel in addition to aspirin and 151 on ticagrelor in addition to aspirin. Majority of patients were discharged on appropriate secondary prevention and there was no significant difference between the two groups (Table 2).

The ticagrelor group had a non-significant trend towards higher rates of revascularization with PCI (57% vs. 48%, *p* = 0.151), possibly reflecting more contemporary practice. Both groups demonstrated a similar moderate risk of bleeding based on the CRUSADE score (10.3% clopidogrel vs. 9.1% ticagrelor, *p* = 0.220). Left ventricular function at discharge was similar in both groups, with the majority observed to have preserved systolic function (52.3% vs. 51.8%, *p* = 1.0).

### 3.2. Clinical Follow-Up

**Figure 1:**
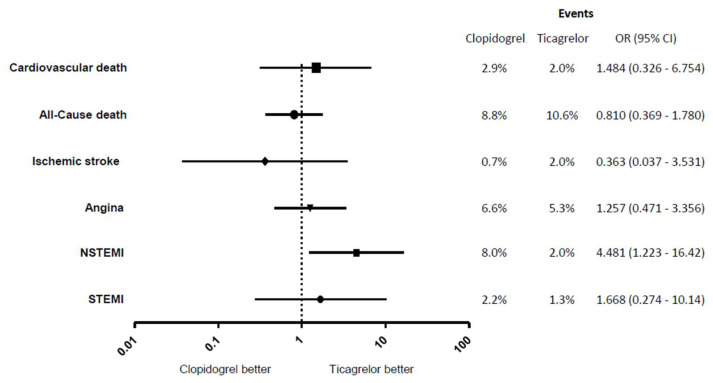
Twelve-month clinical outcomes expressed as odd’s ratio. NSTEMI, non-ST elevation MI; STEMI, ST-elevation MI; OR, odd’s ratio; CI, confidence interval.

A 12-month clinical follow-up was obtained for 288 patients (Figure 1). All patients continued their DAPT for 1 year. There were no significant differences in all-cause mortality (8.8% vs. 10.6%, *p* = 0.69), cardiovascular mortality (2.9% vs. 2.0%, *p* = 0.71), ischemic stroke (0.7% vs. 2.0%, *p* = 0.62), angina (6.6% vs. 5.3%, *p* = 0.80) or STEMI (2.2% vs. 1.3%, *p* = 0.67) between patients discharged on clopidogrel or ticagrelor. Patients treated with clopidogrel, however, had an increased re-admission rate with NSTEMI compared to ticagrelor (8.0% vs. 2.0%, *p* = 0.024, OR 4.481). This is also true for overall myocardial infarction, STEMI and NSTEMI (10.2% vs. 3.3%, *p* = 0.030).

**Figure 2:**
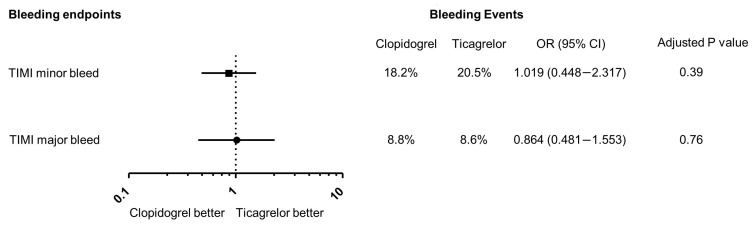
Twelve-month bleeding outcome using TIMI bleeding criteria. OR, odds ratio; CI, confidence interval.

No difference was observed in either major (8.6 vs. 8.8%, *p* = 1.0) or minor TIMI bleeding (20.5% vs. 18.2%, *p* = 0.66) (Figure 2) and following PSM (major bleeding 8.6% vs. 7.0%, *p* = 0.76; minor bleeding 15.7 vs. 22.5%; *p* = 0.39). The lowest median hemoglobin was 108 g/L in the clopidogrel group and 105 g/L in the ticagrelor group (*p* = 0.63). The median decrease in hemoglobin was also similar between the groups (14 g/L vs. 16 g/L, *p* = 0.85). One patient suffered a traumatic sub-dural bleed whilst on ticagrelor but survived to the end of follow-up period.

## 4. Discussion

Ticagrelor is a new P2Y12 inhibitor that is demonstrated to be more predictable, potent and effective compared to clopidogrel [12]. In spite of current guidelines advocating its use, there remains considerable variability with regards to its use in the elderly (≥75) population due to concerns over potential increase in major and life-threatening bleeding.

Our study examined the clinical outcomes of ticagrelor in an unselected contemporary real-world elderly population presenting with acute coronary syndrome. The main finding of our study is that ticagrelor did not cause an increase in major or minor bleeding in the elderly population, consistent with the sub-analysis of PLATO [17]. Our data also suggests ticagrelor may have additional benefits compared to clopidogrel, including a significant reduction in non-ST elevation myocardial infarction (2% vs. 8%, *p* = 0.024) and overall MI (3.3% vs. 10.2%, *p* = 0.030). There was a high incidence of minor TIMI bleeding in both groups (18.2 vs. 20.5%), which reflects the increased bleeding risk in real-world elderly patients who are older and sicker (more presentation with heart failure, more chronic kidney disease and stroke) than the PLATO study population [17]. This is objectively measured with CRUSADE score, which is an established scoring system recommended in ESC guidelines to estimate the risk of bleeding in patients with acute coronary syndrome [7,18,19,20], and the mean score for our cohort of elderly patients was high at (clopidogrel 39 ± 13, ticagrelor 37 ± 13). The event rate for ischemic stroke was low and similar in both groups.

Our findings are consistent with the PLATO trial sub-study that ticagrelor had similar benefit in patient older than 75 and did not increase bleeding compared to clopidogrel [17]. This is also supported by a recent retrospective cohort study which found lower MACE with ticagrelor and no difference in bleeding for elderly patients (>75) with STEMI [13]. Another systematic review and meta-analysis by Zhao et al. in 2021 provided a comparison on the safety and efficacy of ticagrelor in comparison to clopidogrel in the elderly patients admitted with acute coronary syndrome [21]. This was carried out by collecting data from four studies and the total number of participants was 4429. The analysis showed that there was significant reduction in all-cause mortality and cardiovascular death with ticagrelor in comparison to clopidogrel. It did highlight that the bleeding risk did increase with ticagrelor but ticagrelor may well increase longevity in the elderly population [21]. Interestingly, a randomized control trial, POPULAR AGE, demonstrated an increase in bleeding with ticagrelor and no net clinical benefit compared to clopidogrel in patients presenting with NSTEMI [22]. At first glance, the result appeared to be contradictory but there are important differences to consider. Firstly, patients on anti-coagulation were excluded from our study, whereas in POPULAR AGE, more ticagrelor patients were on concurrent anti-coagulation (7% vs. 3%) and evidence suggests triple therapy increases risk of bleeding, especially with ticagrelor [23,24]. Secondly, our study included patients with both STEMI and NSTEMI similar to PLATO while POPULAR AGE only included patients with NSTEMI, and evidence suggests ticagrelor is more effective in STEMI [25,26]. Based on the evidence from PLATO and TRITON-TIMI 38 trials, recent ESC guidelines recommend potent P2Y12 inhibitor (prasugrel or ticagrelor) along with aspirin as standard DAPT in most patients with ACS, and that clopidogrel can be considered as a second-line option in older patients with high bleeding risk [11]. Table 3 summarizes the major studies and trials evaluating ticagrelor in elderly patients.

A significant complication which can impact on the patient outcomes after discharge from hospital is bleeding following an acute coronary syndrome. Bleeding after an acute coronary syndrome has been linked to a higher mortality rate, stroke, recurrent myocardial infarction and repeated admissions to hospital. A study by Kazi et al. in 2015 found that bleeding following discharge from hospital is associated with a mortality hazard ratio almost equivalent to that of a myocardial infarction [27]. In another study by Mehran et al., which was based on data from three large, randomized trials including ACUITY, HORIZON-AMI and REPLACE 2, it was seen that in patients who had bleeding within 30 days of an acute coronary syndrome, the risk of mortality was higher at 1 year [28]. These findings suggest that it is important that appropriate choice of anti-platelets is made, particularly in patients with high risk of bleeding.

The recently published results of the SENIOR-RITA trial showed that in older patients, a routine-invasive strategy did not show benefit over a conservative strategy in terms of cardiovascular death and non-fatal myocardial infarction [5]. This is in contradiction to the FIRE study published in 2023 in patients older than 75 with myocardial infarction and multivessel disease who underwent complete revascularization and had lower rates of death and re-infarction, which was also observed in the subgroup with NSTEMI [29]. The key difference between the two trials is the lack of a control group who did not undergo any revascularization in the FIRE study. Based on the limited evidence and recent advances in interventions, elderly patients with high bleeding risk can at least be treated with a short duration of DAPT [30]. In view of the benefit in the large trials, our study suggests ticagrelor should not be withheld on the basis of age as a perceived risk factor for bleeding in ACS and should be considered as first-line therapy in this age group, especially in high-risk patients such as STEMI.

There are a number of limitations that should be considered when interpreting these observations. This study was small, single-centered, retrospective and not randomized. There could be potential bias due to temporal change in practice, as reflected by the non-significant increased revascularization in the ticagrelor group. The final decision of anti-platelets was with the physician, so there could be potential “physician bias”, although, again, this would reflect ‘real-world’ practice.

**Table 3:** Summary of key registry studies and trials evaluating ticagrelor in elderly ACS patients. CV—cardiovascular; MI—myocardial infarction; MACCE—Major Adverse Cardiac and Cerebrovascular Events; RCT—randomized controlled trial; STEMI—ST-elevation myocardial infarction.

Study	Country/Region	Population	Ticagrelor Use and Comparator	Bleeding Risk	Prognostic Benefit
RENAMI/BleeMACS [31]	Global/Europe	≥75 years	Ticagrelor vs. Clopidogrel	Non-significant increase in bleeding (HR 1.49)	Yes—↓ recurrent MI, ↓ all-cause mortality
SWEDEHEART [32]	Sweden	≥80 years	Ticagrelor vs. Clopidogrel	Increased major bleeding (HR 1.48)	No—↑ all-cause mortality; net clinical harm
Bremen STEMI Registry [13]	Germany	≥75 years	Ticagrelor vs. Clopidogrel	No difference in bleeding after one year (HR 1.00)	Yes—↓ MACCE, improved net clinical benefit
PLATO (Subgroup) [17]	Multinational RCT	≥75 years	Ticagrelor vs. Clopidogrel	No significant increase in major bleeding (HR 1.02)	Yes—↓ CV death, MI, stroke
POPular AGE [22]	Netherlands RCT	≥70 years	Clopidogrel vs. Ticagrelor/Prasugrel	Clopidogrel: Lower major bleeding (HR 0.57)	No difference in ischemic events

## 5. Conclusions

In summary, this study suggests that treatment with ticagrelor was reasonable and did not increase major and life-threatening bleeding events when compared to clopidogrel in elderly patients

## Figures and Tables

**Table 1:** Baseline characteristics. Age, gender, BMI and creatinine are expressed as mean ±standard deviation. Cardiovascular risk factors are expressed as percentage of the total. BMI, body mass index; MI, myocardial infarction; HTN, hypertension; PVD, peripheral vascular disease; CKD, chronic kidney disease; PCI, percutaneous coronary intervention; CABG, coronary artery bypass grafting; CAD, coronary artery disease. *p* < 0.05 is considered statistically significant.

		Clopidogrel (137)	Ticagrelor (151)	*p* Value
Age		80 ± 5	81 ± 5	0.062
Gender	Male	56%	53%	0.722
	Female	44%	47%	
BMI		24 ± 8	26 ± 4	0.953
Creatinine		108 ± 74	105 ± 85	0.806
Cardiovascular risk factors				
	Previous MI	29.9%	30.5%	1.000
	Angina	29.2%	31.8%	0.701
	Hypertension	73.7%	72.8%	0.895
	Hypercholesterolemia	42.3%	47.0%	0.477
	PVD	5.1%	6.6%	0.626
	Stroke	21.2%	20.5%	1.000
	CKD	13.9%	18.5%	0.339
	Heart failure	13.9%	11.9%	0.601
	Previous PCI	16.8%	17.9%	0.877
	Previous CABG	12.4%	13.9%	0.731
	Family history of CAD	15.3%	16.6%	0.872
Smoker	Current	11.4%	7.9%	0.422
	Ex-Smoker	40.2%	33.8%	0.393
Diabetes	Insulin depedent	10.9%	10.0%	0.848
	Non-insulin	16.8%	19.9%	0.545

**Table 2:** Presentation, management, crusade score, discharge LV function and medications presented as percentage of the total. STEMI, ST-elevation myocardial infarction; NSTEMI, non-ST elevation myocardial infarction; PCI, percutaneous coronary intervention; LV, left ventricle; ACE, angiotensin converting enzyme. *p* < 0.05 is considered statistically significant.

		Clopidogrel (137)	Ticagrelor (151)	*p* Value
Presentation	STEMI	31.4%	43.1%	0.051
	NSTEMI	68.6%	56.9%	
ECG changes	ST elevation	27.7%	37.1%	0.100
	ST depression	24.1%	19.9%	0.259
	T wave changes	18.2%	18.5%	1.000
	Left bundle branch block	3.6%	6.0%	0.420
	Other acute changes	11.7%	10.6%	0.852
	No acute change	13.9%	7.9%	0.123
	Unknown	0.7%	0	
Kilip class	≥3	11.7%	10.6%	0.852
Infarction territory	Anterior	25.5%	33.8%	0.156
	Lateral	3.6%	6.0%	0.420
	Inferior	21.2%	24.5%	0.575
	Posterior	2.9%	2.6%	1.000
	Indeterminate	11.7%	11.3%	1.000
	Unknown	35.0%	21.9%	0.018
Treatment	Primary PCI	23.4%	31.7%	0.116
	PCI	48.1%	57.0%	0.157
	Medical Management	51.9%	43.0%	
Crusade score		39 ± 13	37 ± 13	0.220
Discharge LV function	Good	51.8%	52.3%	1.000
	Moderately impaired	25.5%	29.8%	0.433
	Poor	6.6%	7.3%	1.000
	Not assessed	16.1%	10.6%	0.222
Discharge Medications	Statin	88.3%	92.7%	0.228
	ACE inhibitor	74.4%	79.1%	0.489
	Beta-blocker	78.8%	80.1%	0.779

## Data Availability

All data is contained within the article/Supplementary Materials. Further inquiries can be made to the corresponding author.

## Supplementary Materials

The following supporting information can be downloaded at: https://www.mdpi.com/article/10.3390/jcm14165629/s1, Figure S1: CONSORT flow chart; Table S1: Baseline characteristic after propensity score matching; Table S2: Presentation, management, crusade score, discharge LV function and medications after propensity score matching.
