# Supplementary material for: Safety of Ticagrelor Compared to Clopidogrel in the Contemporary Management Through Invasive or Non-Invasive Strategies of Elderly Patients Presenting with Acute Coronary Syndromes
Source: J Clin Med. 2025 Aug 8;14(16):5629. doi: 10.3390/jcm14165629 (PMC12386568; doi:10.3390/jcm14165629)
Supplement: Supplementary file 1 [file jcm-14-05629-s001.zip › jcm-3722723-supplementary.pdf]

Table S1

Baseline characteristics after propensity score matching. BMI; body mass index; MI; myocardial infarction; HTN, hypertension; PVD, peripheral vascular disease; CKD, chronic kidney disease; PCI, percutaneous coronary intervention; CABG; coronary artery bypass grafting; CAD, coronary artery disease. $p<0.05$ is considered statistically significant.

		Clopidogrel (70)	Ticagrelor (71)	p value
**Age**		80±4	80±4	0.67
**Gender**	Male	59%	59%	1.00
	Female	41%	41%	
**BMI**		26±5	26±4	0.72
**Creatinine**		109±90	116±115	0.71
**Cardiovascular risk factors**				
	Previous MI	28.6%	35.2%	0.47
	Angina	24.8%	35.2%	0.20
	Hypertension	72.8%	77.5%	0.56
	Hypercholesterolaemia	38.6%	52.1%	0.14
	PVD	4.3%	2.8%	0.68
	Stroke	21.4%	21.2%	1.00
	CKD	15.7%	22.5%	0.39
	Heart failure	14.3%	14.1%	1.00
	Previous PCI	12.8%	25.3%	0.09
	Family history of CAD	14.3%	19.7%	0.08
**Smoker**	Current	12.8%	7.1%	0.53
	Ex-Smoker	37.1%	40.8%	0.52
**Diabetes**	Insulin depedent	10.0%	14.1%	0.39
	Non-insulin	14.3%	16.9%	0.81

Table S2

Presentation, management, crusade score, discharge LV function and medications after propensity score matching. STEMI; ST-elevation myocardial infarction; NSTEMI; Non-ST elevation myocardial infarction, PCI; percutaneous coronary intervention; LV, left ventricle., ACE – angiotensin converting enzyme. P<0.05 is considered statistically significant.

		Clopidogrel (70)	Ticagrelor (71)	p value
**Presentation**	STEMI	42.8%	39.4%	
	NSTEMI	57.2%	60.6%	0.73
**ECG changes**	ST elevation	35.7%	29.6	
	Left bundle branch block	7.1%	9.8%	0.93
	ST depression	15.7%	18.3%	
	T wave changes	17.1%	16.9%	
	No acute change	24.3%	25.3%	
**Infarction territory**	Anterior	24.3%	33.8%	
	Inferior	28.6%	23.9%	
	Lateral	1.4%	5.6%	0.24
	Posterior	1.4%	1.4%	
	Indeterminate	10.0%	15.5%	
	Unknown	34.3.0%	19.7%	
**Treatment**	Primary PCI	47.2%	38.0%	
	PCI	52.8	60.6%	0.36
	Medical Management	0.0%	1.4%	
**Crusade score**		38±13	37±13	0.59
**Discharge LV function**	Not assessed	14.3%	8.4%	
	Good	57.1%	46.6%	
	Moderate	18.6%	35.2%	0.21
	Poor	8.6%	7.0%	
	Unknown	1.4%	2.8%	
**Discharge Medications**	Statin	90.0%	90.1%	1.00
	ACE inhibitor	714%	776.0%	0.57
	Beta-blocker	75.7%	85.9%	0.14

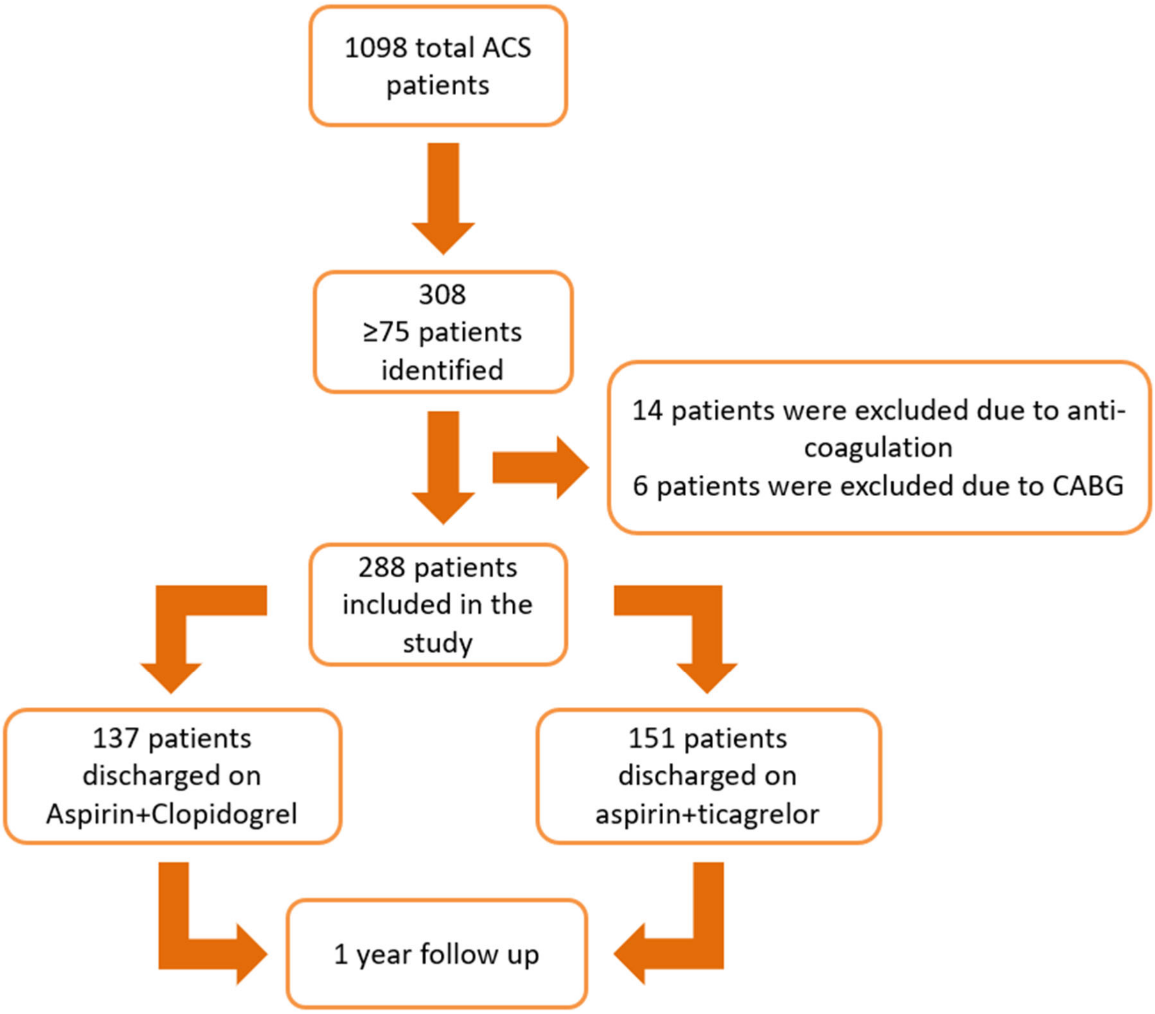

Figure S1. CONSORT flow chart.
